# Antibiotic envelope is associated with reduction in cardiac implantable electronic devices infections especially for high‐power device—Systematic review and meta‐analysis

**DOI:** 10.1002/joa3.12270

**Published:** 2019-11-29

**Authors:** Raymond Pranata, Alexander Edo Tondas, Rachel Vania, Yoga Yuniadi

**Affiliations:** ^1^ Faculty of Medicine Universitas Pelita Harapan Tangerang Indonesia; ^2^ Department of Cardiology and Vascular Medicine Faculty of Medicine Universitas Sriwijaya Dr. Mohammad Hoesin General Hospital Palembang Indonesia; ^3^ Department of Cardiology and Vascular Medicine, Faculty of Medicine Universitas Indonesia National Cardiovascular Center Harapan Kita Jakarta Indonesia

**Keywords:** antibiotic envelope, cardiac implantable electronic device, infection, mortality, TYRX

## Abstract

**Background:**

Infections after cardiac implantable electronic device (CIED) placement are associated with significant morbidity and mortality. The incidence of CIED is increasing overtime despite the optimal use of antimicrobial agents. This systematic review and meta‐analysis will address the latest evidence on the use of AE to mitigate the risk of CIED infection, and which subset of patients will they benefit the most.

**Methods:**

We performed a comprehensive search on topics that assesses antibiotic envelope and implantable cardiac electronic device up until August 2019.

**Results:**

There were a total of 32,329 subjects from six studies. Antibiotic envelope was associated with a lower risk of major infection with OR 0.42 [0.19, 0.97], *P* = .04; I^2^: 58% and HR 0.52 [0.32, 0.85], *P* = .009; I^2^: 80%. Upon sensitivity analysis by removing a study, the OR became 0.40 [0.27, 0.59], *P* < .001; I^2^: 46%. Subgroup analysis for 12 months’ infection was OR 0.65 [0.43, 0.99], *P* = .04; I^2^: 49%. Meta‐analysis of propensity‐matched cohort showed a reduced risk of infection with AE (OR of 0.14 [0.05, 0.41], *P* < .001; I^2^:0%). Mortality was similar in both AE and control groups. Antibiotic envelope reduced the incidence of infection in patients receiving high‐power device (OR 0.44 [0.27, 0.73], *P* = .001; I^2^:0%) but not low‐power device.

**Conclusion:**

Antibiotic envelope (TYRX) was found to be safe and effective in reducing the risk of major infections in high‐risk patients receiving CIED implantation, especially in those receiving high‐power CIED.

## Graphical abstract

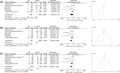

## 1 INTRODUCTION

Cardiac implantable electronic device (CIED) is commonly used worldwide by cardiac electrophysiologists to treat various cardiovascular problems including cardiomyopathies, rhythm disorders, and prevention of sudden cardiac deaths.^1^, ^2^, ^3^ Infections after CIED placement are associated with significant morbidity and mortality.^4^, ^5^, ^6^ The incidence of CIED is increasing overtime despite the optimal use of antimicrobial agents.^7^, ^8^, ^9^

Antibiotic envelope (AE) (TYRX Absorbable Antibacterial Envelope) was developed to mitigate the risk of infection.^10^ TYRX is an absorbable, multifilament mesh envelope that was cleared by Food and Drug Administration in the year 2008 (old generation) and 2013 (new generation). It has been shown to be useful in the preclinical and clinical study.^10^, ^11^, ^12^ Although there are studies that associate the use of envelope to the increased risk of infection and mortality.^13^, ^14^ Recently, the results of the first randomized trial was published, which adds to the body of literature (mostly retrospective cohorts). However, it is still unclear whether AE would be beneficial and safe in all patients receiving CIED, and whether they apply to all subset of patients only. This systematic review and meta‐analysis will address the latest evidence on the use of AE to mitigate the risk of CIED infection, and which subset of patients will they benefit the most.

## 2 METHODS

### 2.1 Search strategy

**Figure 1:**
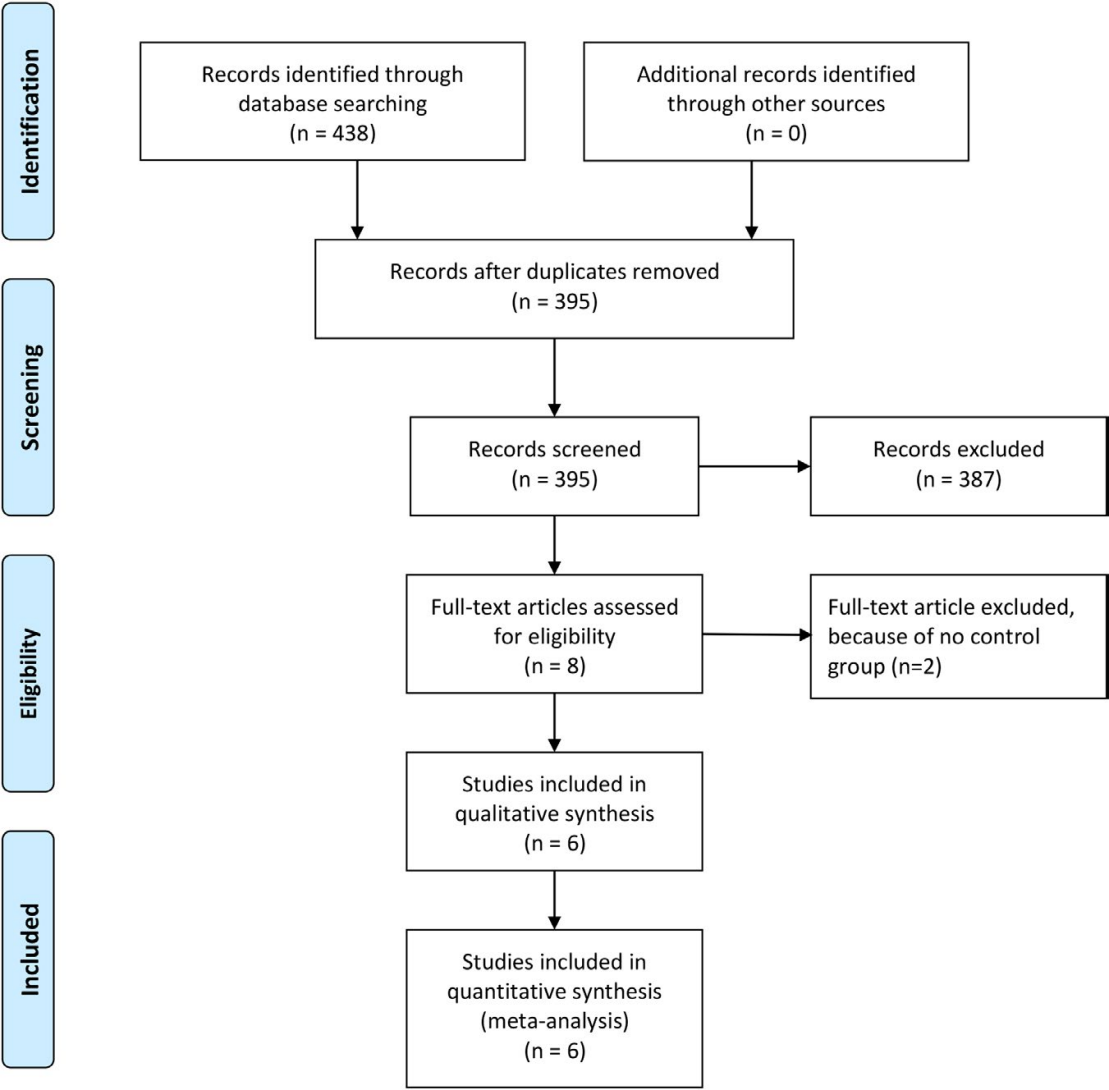
Study flow diagram

We performed a comprehensive search on topics that assesses AE and CIED including implantable cardioverter‐defibrillator (ICD), permanent pacemaker (PPM), and cardiac resynchronization therapy (CRT) including CRT‐pacemaker (CRT‐P), and CRT‐defibrillator (CRT‐D) with keywords [“antibiotic envelope” and “cardiac device”] and its synonym from inception up until August 2019 through PubMed, EuropePMC, Cochrane Central Database, and hand‐sampling from potential articles cited by other studies. The records were then systematically evaluated using inclusion and exclusion criteria. We also perform hand sampling from references of the included studies. Two researchers (R.P and R.V) independently performed an initial search, discrepancies were resolved by discussion. A Preferred Reporting Items for Systematic Reviews and Meta‐Analyses flowchart of the literature search strategy of studies was presented in Figure 1.

### 2.2 Selection criteria

The inclusion criteria for this study are all studies that assess antibiotic envelope and CIED. We include all related clinical researches/original articles and exclude case reports, review articles, and non‐English language articles.

### 2.3 Data extraction

Data extraction and quality assessment were done by two independent authors (R.P and AET) using standardized extraction form which includes authors, year of publication, study design, subject characteristics, sample size, type of antibiotic envelope, major infections, mortality, and follow‐up duration.

### 2.4 Statistical analysis

To perform the meta‐analysis, we used RevMan version 5.3 software (Cochrane Collaboration). We used the odds ratio (OR) and a 95% CI as a pooled measure for dichotomous data. We used hazard ratio (HR) for the pooled measure of time‐to‐event analysis. Inconsistency index (I^2^) test, which ranges from 0% to 100%, was used to assess heterogeneity across studies. A value above 50% or *P* < .05 indicates statistically significant heterogeneity. We used the Mantel‐Haenzsel method for OR and inverse variance for HR with a fixed effects model for meta‐analysis, and a random effects model was used in case of heterogeneity if appropriate. All P values were two‐tailed with a statistical significance set at 0.05 or below. We also perform sensitivity analysis and subgroup analysis whenever possible/appropriate.

## 3 RESULTS

We found a total of 438 results on the initial search. There were 395 records after removal of duplicates. Three hundred and eighty‐seven records were excluded after screening the title/abstracts. After assessing eight full‐text for eligibility; we excluded two because of no control group. We included six studies in qualitative synthesis and meta‐analysis. (Figure 1) Five studies were cohort, and one was a randomized controlled trial. There were a total of 32,329 subjects from six studies.^12^, ^13^, ^14^, ^15^, ^16^, ^17^ (Table 1).

**Table 1:** Summary of the included studies

Author	Study design	Patient characteristics	Sample (n)	Antibiotic envelope (n)	Antibiotic envelope type	Age (years ± SD)	Male (%)	Major infection	Mortality	Follow‐up
Tarakji (WRAP‐IT) 2019	Randomized controlled trial	Patient at increased risk for CIED infection	6983	3495/3488	TYRX	70.05 ± 12.5	71.7	25(0.72)/ 42(1.2)	349(10)/ 365(10.5)	20.7 ± 8.5 months
Henrikson (Citadel/Centurion) [Medicare Control] 2017	Multicenter prospective cohort	>18 years of age undergoing CIED replacement with an ICD (Citadel) or CRT (Centurion)	22 012	578/21434	TYRX	N/A	N/A	4 (0.7)/ 285(1.3)	64(11)/2873(13.4	12 months
Hassoun 2017	Retrospective cohort	Patient undergoing CIED implantation	184	92/92	AIGIS_Rx_ (TYRX)	69/73	60	5(5.4)/ 1(1.08)	2(2.2)/0(0)	9 months
Kolek 2015	Retrospective cohort	≥2 risk factors for CIED infection	1124	488/636	TYRX (353)/ TYRX‐A(135)	Median 67‐70	65.3	1(0.2)/ 20(3.1)	N/A	569 days (524‐640) & 559 days (435‐768)
Shariff 2015	Retrospective cohort	Every patient undergoing a CIED procedure in the electrophysiology laboratory	1476	365/1111	AIGIS_Rx_ (TYRX)	70 ± 15.2	64.8	0(0)/ 19(1.7)	15(4.1)/74(6.7)	6 months
Mittal 2014	Retrospective cohort	Patient at high risk for CIED infection	550	275/275	AIGIS_Rx_ (TYRX)	74.5 ± 11.5	74.2	3(1.1)/ 10(3.6)	N/A	6 months

Abbreviations: CIED = Cardiac Implantable Electronic Devices; CRT = Cardiac Resynchronization Therapy; ICD = Implantable Cardioverter Defibrillator; N/A = Not Applicable/Available; WRAP‐IT = Worldwide Randomized Antibiotic Envelope Infection Prevention Trial

### 3.1 Characteristics of included studies

Most of the studies included patients that were at risk for CIED infection. Henrikson et al study provides two alternative controls (Medicare control and Centurion control), we included both in meta‐analysis. 5,293 patients received AE, and 27,036 did not in Medicare control scenario. In Centurion control scenario; 5,293 patients received AE and 25,471 did not. All of the studies included used TYRX, and one of the studies used TYRX‐A. AIGIS_Rx_ was same as TYRX. The patients’ age was around 70 years old. Major infection was 38 (0.72%) in AE group and 377 (1.4%) in control group (Medicare scenario). In Centurion scenario, major infection was 38 (0.72%) and 98 (1.59%) in control group. Follow‐up differs between studies; however, the follow‐up for infection outcome included in this meta‐analysis was 6 months (Shariff et al; Mittal et al), 300 days (Kolek 2015), and 12 months (Hassoun et al; Tarakji et al; Henrikson et al).

### 3.2 Major infection

**Figure 2:**
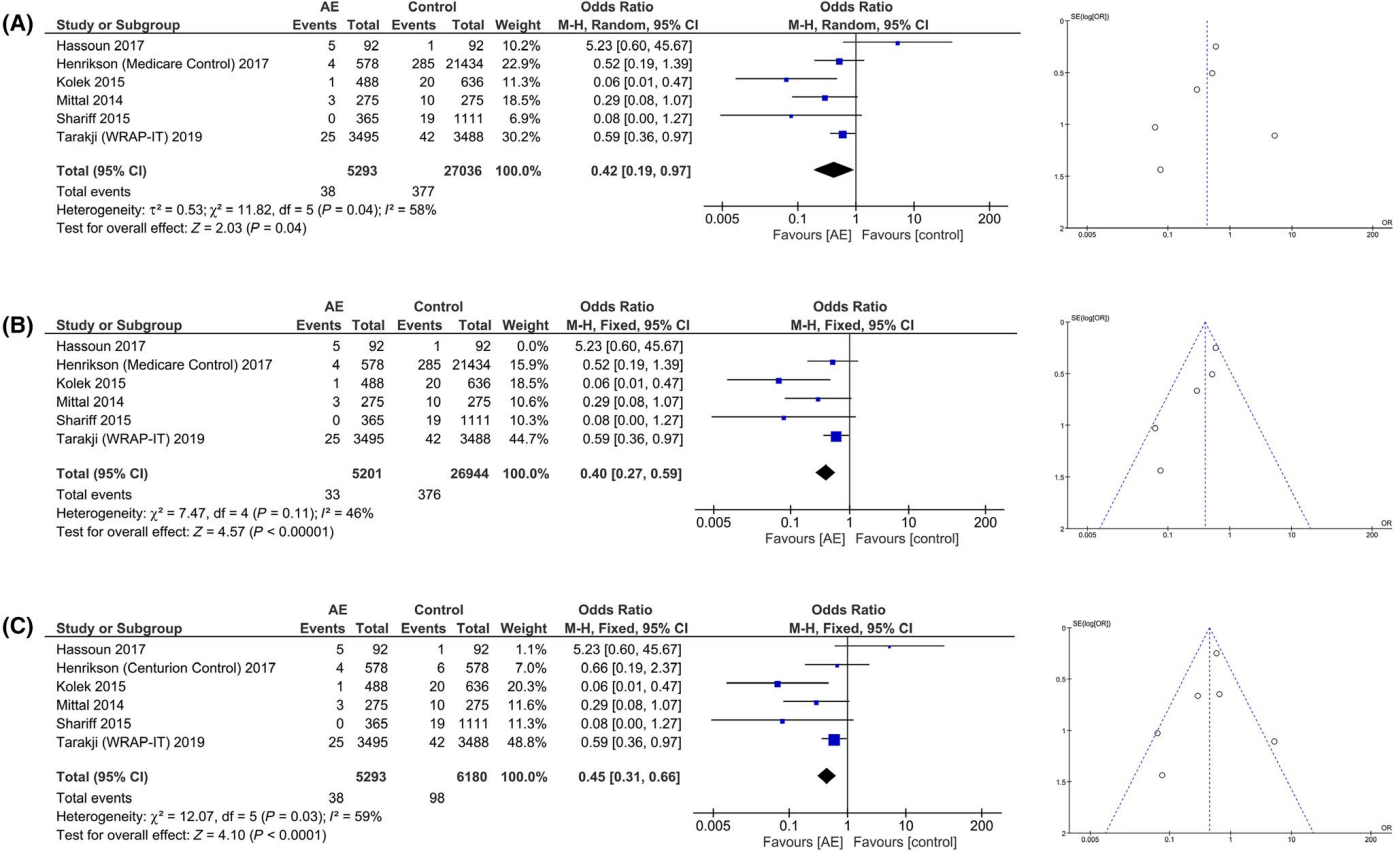
Antibiotic envelope and major infections after CIED implantation. A, showed a decreased incidence of major infections in group receiving antibiotic envelope. B, showed that heterogeneity can be reduced by removing a study. C, is a forest plot with Centurion as control for Henrikson et al study; fixed effects model showed significant result but not random effects model. Corresponding funnel plot showed that risk of publication bias cannot be neglected

Five studies showed that AE reduces the risk of infection; however, one study showed an increased risk of infection in the AE group. On meta‐analysis using M‐H formula, that only two studies individually have significant odds ratio. Pooled analysis showed OR 0.42 [0.19, 0.97], *P* = .04; I^2^:58%, *P* = .04 [Figure 2A] for the risk of infection in the AE group. Upon sensitivity analysis by removing a study, we found that upon removal of Hassoun et al study, the OR became 0.40 [0.27, 0.59], *P* < .001; I^2^:46%, *P* = .11 [Figure 2B]. Antibiotic envelope was associated with a decreased HR of 0.52 [0.32, 0.85], *P* = .009; I^2^:80%, *P* = .03 for infection [Figure 2C]. Subgroup analysis for 12 months’ infection was OR 0.65 [0.43, 0.99], *P* = .04; I^2^:49%, *P* = .14.

In the second scenario, we select Centurion control for Henrikson et al study. On meta‐analysis we found that AE was associated with OR 0.45 [0.31, 0.66], *P* < .001; I^2^:59%, *P* = .03 for the risk of infection. Random effects model yields a nonsignificant result. The risk of bias, as shown by funnel‐plot analysis was moderate‐high. Propensity‐matched analysis was present in three studies, and a pooled analysis showed an OR of 0.14 [0.05, 0.41], *P* < .001; I^2^:0%, *P* = .38.

### 3.3 Mortality

**Figure 3:**
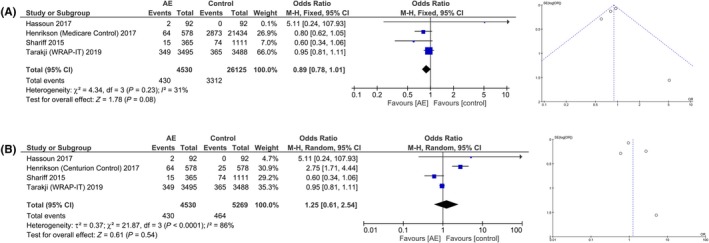
Antibiotic envelope and mortality after CIED implantation. A, showed that there is no statistically significant difference in mortality between the two groups. B, is a forest plot with Centurion as control for Henrikson et al study. Corresponding funnel plot showed that risk of publication bias was high

The risk of mortality did not differ between AE group and the control group in both Medicare [Figure 3A] and Centurion control scenario [Figure 3B] for Henrikson et al study in both random and fixed effects model. Sensitivity analysis did not change the outcome in both scenarios. There was no difference in HR between AE and control groups. The corresponding funnel‐plot showed that the risk of publication bias was high.

### 3.4 High‐power and Low‐power device

**Figure 4:**
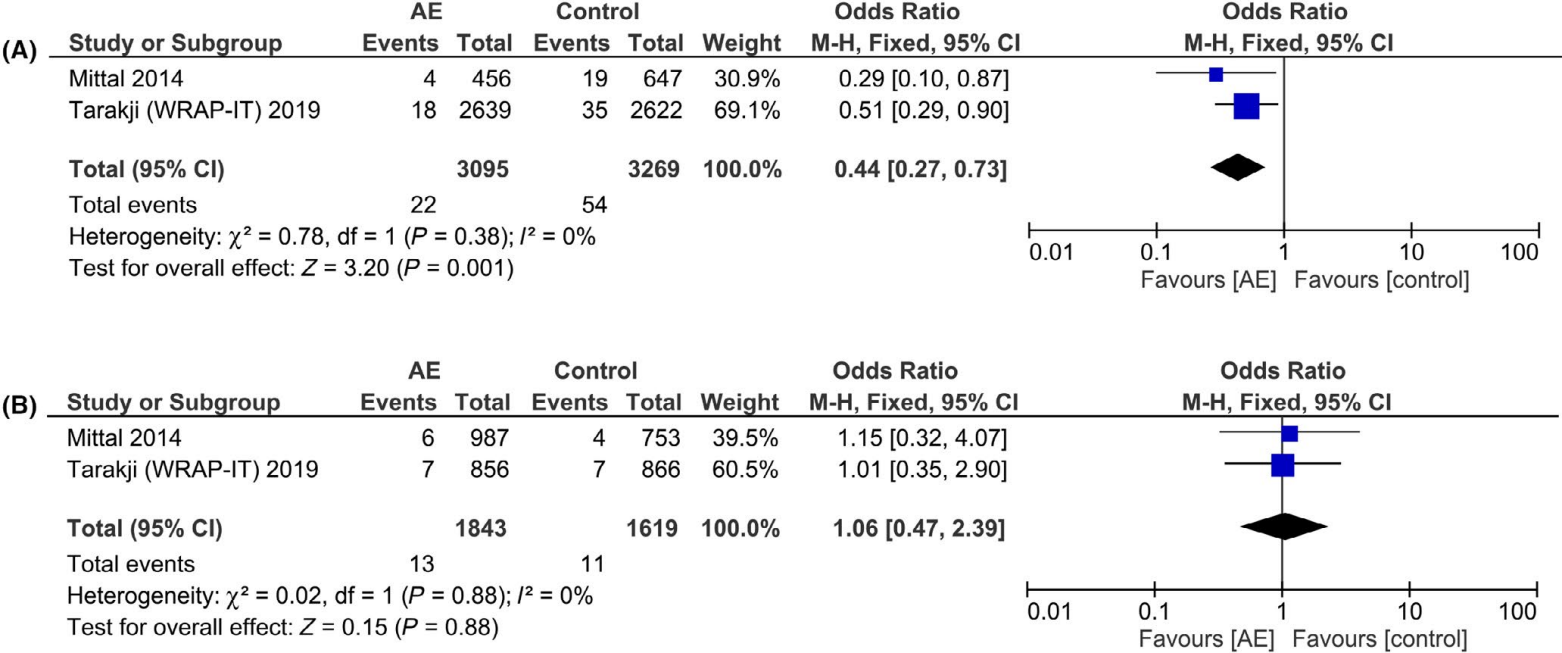
Subgroup analysis on high‐power and low‐power device. Antibiotic envelope was associated with a lower incidence of major infection in patients receiving high‐power device (A) but not low‐power device (B)

In subgroup analysis, we explore the effectiveness of AE on patients receiving high‐power device (CRT‐D and ICD) and low‐power device (CRT‐P and pacemaker) placement. The incidence of infection in the high‐power device was 22 (0.71%) in AE group and 54 (1.65%) in the control group (statistically significant). The incidence of infection in low‐power device was 13 (0.71%) in AE group and 11 (0.68%) in control group (statistically not significant). Subgroup analysis showed that AE reduced the incidence of infection in patients receiving high‐power device (OR 0.44 [0.27, 0.73], *P* = .001; I^2^:0%, *P* = .37) [Figure 4A]. Antibiotic envelope did not reduce the risk of infection in patients undergoing low‐power device implantation (OR 1.06 [0.47, 2.39], *P* = .88; I^2^:0%, *P* = .88) [Figure 4B].

## 4 DISCUSSION

This systematic review and meta‐analysis showed that the use of AE (TYRX) was associated with a reduced rate of major infections, especially in patients receiving high‐power CIED. Mortality was similar in both AE and control groups. The risk of publication bias remained high, as shown in the funnel‐plot analysis.

About 70% of the CIED infection is caused by *Staphylococcal* species in which approximately 50% of this infection is attributed to the methicillin‐resistant organism and the rest comprised of mostly gram‐negative bacterias and negative bacterial culture infection.^12^, ^18^ Absorbable single‐use AE (TYRX) dissipates rifampin and minocycline which increase the antibiotic concentration in local tissue for over than 7 days while stabilizing CIED. TYRX is shown to be effective for various types of bacteria, including *Staphylococcus epidermidis* in preclinical and clinical studies*.* 10, 11 A new generation of TYRX is fully absorbed in approximately 9 weeks.^16^ Unfortunately, most of the studies included in this meta‐analysis used an older generation of TYRX.

There was one randomized controlled trial in the study, namely WRAP‐IT trial. The trial has a low risk of bias when evaluated using the Cochrane risk‐of‐bias tool for randomized trials. The result of this trial is in accordance with most of the previous studies, which showed that AE reduced incidence of major infections along with reliable safety endpoint (no difference in mortality). The cause of heterogeneity based on sensitivity analysis was Hassoun et al study; and they have a dissenting finding that was probably because of the differences in baseline characteristics. The authors of the study admitted that there was a difference in baseline characteristics, and their sample size was small. The most striking difference was the percentage of replacement/revision of CIED was 51% in AE group vs only 9% in control group. Replacement/revision of CIED was known to increase the risk of major CIED infection by twofold.^19^ We were unable to analyze how significant was this factor in determining the outcome for the intervention and control group, whether the hazard of AE is still significant after adjustment with the other factors. However, it seemed that the difference majorly influenced the result. We found that in a pooled analysis of three propensity‐matched cohorts, AE was strongly associated with reduced risk of infection.^12^, ^15^, ^17^ Moreover, the randomized controlled trial also showed that AE reduced the incidence of infection.^16^ Based on this consideration, we selected a fixed effects model for some pooled analysis even though the heterogeneity was above 50%.

The lack of impact on the mortality by the use of AE is possibly because of the mortality in these studies refers to all‐cause mortality and its emphasis on safety outcome rather than the primary outcome. WRAP‐IT trial demonstrates that the mortality is similar in both groups, however, one of the component of their primary outcome (major CIED infection) includes death caused by CIED infection.^16^ The number of deaths caused by CIED infection in AE group vs control group is unclear; however, the primary outcome was demonstrated to be lowered in the AE group. The mortality in this study is a safety outcome. Shariff et al study showed that there is no infection in AE group compared to 1.7% in the control group and infection were associated with higher 6‐month mortality (15.7% vs 4.5%, *P* = .021).^15^ In Hassoun et al study, the AE group has more risk factors compared to the control group, and such difference may negate the effect of AE.^14^ From these observations it seemed that AE is associated with lower infection‐associated mortality but not all‐cause mortality. To the best of our knowledge, there was no study reporting the difference of period after implantation to infection diagnosis between AE vs control group (there are few studies that report time to infection in general. As for the duration of hospitalization for infection, there were no separate data on antibiotic envelope vs control group; there was one study that reported a longer LOS in antibiotic envelope group, the cause of hospitalization was not clearly defined, but it seemed that the length of stay is not specific to infection.

Several factors have been shown to increase the risk of CIED infection, including abdominal pocket, epicardial leads, positioning of two or more leads, dual‐chamber systems reintervention for lead dislodgement, device replacement/revision, lack of antibiotic prophylaxis, temporary pacing, inexperienced operator, and procedure duration increase the risk of device infection.^19^ Hence, these factors were the consideration for performing subgroup analysis on device type; unfortunately, we were unable to perform analysis in ICD, CRT, and PPM individually because of the lack of data; we can only perform a meta‐analysis for high‐power vs low‐power device. Implantation of ICD or CRT‐D (high‐power device) was shown to be an independent predictor of CIED infection which is possibly because of the more prolonged procedure, multiple leads, higher incidence of early re‐intervention for LV lead complications, and higher comorbidities.^20^, ^21^, ^22^ In this meta‐analysis, we found that the risk reduction was more pronounced in high‐power device; a plausible explanation is because of the implantation of low‐power device has a lower risk of major infections compared to high‐power device and the benefit was not observed because of the small number of events in both AE and control group in low‐power device.^12^, ^16^ From this observation, the rate of major CIED infection is low enough that it does not warrant AE use in low‐power device except in special cases.

In patients at risk of major infection, the use of AE to prevent infections did not increase the cost significantly and is economically reasonable. Shariff et al showed that by extrapolating the infection rate and costs observed in the AE group to the control group, 6.2 additional infections in the control group costs approximately $340,000 compared to the actual cost of device in AE group estimated at $320,000. 15 However, the initial cost of using antibiotic envelope can be burdensome especially in low‐ to middle‐income countries.

The clinical implication of the finding of this study is that the adjunctive AE is beneficial to prevent major infection in patients receiving CIED compared to standard therapy alone. Considering the cost of AE, it is preferable to use AE only in a situation perceived to have profound benefit, especially if the patient has financial constraint. Since the benefit seemed to be negligible in patients receiving low‐power device (with the current evidence), it is advisable to use AE only on patients receiving high‐power device unless there are other factors that may potentially increase the risk of infection in low‐power device implantation.

Limitation in this systematic review includes selection bias where negative finding research is less likely to be published; this is further reflected by the asymmetrical funnel‐plot analysis. Many of the studies were retrospective studies, and there was only one randomized controlled trial. The prospective cohort study lacked a control arm native to the study. The subgroup analysis for the device was only feasible for high‐power and low‐power device; it cannot be performed in PPM, ICD, and CRT individually. Also, the number of study with high‐power vs low‐power device was limited. The total events for low‐power device subgroup meta‐analysis were low, which means that the benefit of AE in these populations cannot be ruled out. Most of the studies did not perform multivariate analysis on predictors of infection. Possibly because of the small number of events and model overfitting that may result if such analysis is performed. There was also lack of data on the therapy of CIED infections. WRAP‐IT trial defined major CIED infection as infections that resulted in CIED system removal, an invasive CIED procedure (eg, pocket revision without removal), treatment with long‐term antibiotic therapy (if the patient was not a candidate for system removal) with infection recurrence after discontinuation of antibiotic therapy, or death. However, to the best of our knowledge, the included studies did not provide the proportion of subjects receiving CIED removal, pocket revision without removal or long‐term antibiotic as intervention for the major CIED infection.

## 5 CONCLUSION

Antibiotic envelope (TYRX) was found to be safe and effective in reducing the risk of major infections in a high‐risk patient receiving CIED implantation, especially in those receiving high‐power device (CRT‐D and ICD).

## CONFLICT OF INTEREST

The authors declare that they have no conflict of interests.

## AUTHORS CONTRIBUTION

Raymond Pranata conceived and designed the study and drafted the manuscript. Raymond Pranata and Rachel Vania acquired the data and drafted the manuscript. Raymond Pranata and Alexander Edo Tondas interpreted the data and performed extensive research for the manuscript. Yoga Yuniadi critically revise the manuscript. All authors contributed to the writing of the manuscript. Raymond Pranata performed the data analysis.
